# NETosis and SARS-COV-2 infection related thrombosis: a narrative review

**DOI:** 10.1186/s12959-022-00375-1

**Published:** 2022-03-30

**Authors:** Mahin Behzadifard, Masoud Soleimani

**Affiliations:** 1 Dezful University of Medical Sciences, Dezful, Iran; 2 Department of hematology, Tarbiat Modares University, Tehran, Iran

**Keywords:** SARS-CoV-2, COVID-19, Thrombosis, Tissue factor, Angiotensin converting enzyme, NETosis, NETs, Factor seven activating protease (FSAP), Tissue factor pathway inhibitor, TFPI, Acute respiratory distress syndrome, ARDS

## Abstract

**Background:**

Coronavirus disease 2019 (COVID-19) infection is related to immune hyperactivity, the release of inflammatory cytokines, and immunothrombosis. Among the underlying mechanisms in COVID-19 thrombosis, neutrophil extracellular traps (NETs) formation, NETosis, may have a significant role. COVID-19 thrombi obtained from extracorporeal membrane oxygenation contained an accumulation of neutrophils and in a higher amount of NETs when compared with non-COVID-19 thrombi specimens.

**Main body:**

During sepsis and inflammatory status, NETs released from neutrophils and histones and nucleosomes extruded into the extracellular space and take part in the host innate immunity defense, inflammation, and thrombosis. Excessive NETosis is related to clinical progression and respiratory failure in infections and sepsis. NETosis act as a scaffold for thrombus formation, and new associative data support the relation between deregulated immune responses with thrombus formation and organ failure. NETosis is reported in COVID-19 patients. In COVID-19 infection, overproduction of tissue factor (TF) by neutrophils has a role in immunothrombosis. Additionally, NETs can trap TF pathway inhibitor (TFPI) as the only endogenous protein that effectively inhibits the activity of the significant proteases– complexes, TF–FVIIa and prothrombinase.

**Conclusion:**

Because of NETosis can induce intrinsic and extrinsic coagulation cascade activation through the production of TF, activation of FXII, and inhibition of TFPI and fibrinolysis and induce immunothrombosis, targeting NETosis may diminish thrombus formation related to NETs in COVID-19 patients.

## Introduction

The pathophysiology of COVID-19 infection-related thrombosis is still poorly understood. Endothelial damage and subsequently activation of coagulation cascade can lead to widespread microvascular thrombi formation in the lungs and other organs. The lungs injury increases alveolar and capillary permeability that then will be shown as ground-glass appearance in the chest X-ray, and clearly ARDS [1]. Elevated evidence has been shown an increased risk of thrombosis in severe COVID-19 patients and suggested microvascular thrombosis as a main pathophysiologic parameter in COVID-19 infection [2, 3]. The complement system, inflammatory cytokines, and neutrophil extracellular traps (NETs) formation, NETosis, can contribute to coagulation activation [4, 5]. Various pathogens such as bacteria, fungi, protozoa, and viruses can activate NETosis. Cytokines and chemokines like IL-8 and TNF, antibodies and immune complexes, and microcrystals can induce NETs formation [6–8].

NETs are actively released from neutrophils into the extracellular space [9]. NETs formation is a part of innate immunity and is described as responsible for trapping the pathogens, killing the microbes, and inducing inflammation [10]. The NETosis contributes to sepsis and acute respiratory distress syndrome(ARDS) pathogenesis and causes vascular tissue damage, thrombosis, multiorgan failure, and death [11, 12]. Increased NETs formation correlates with COVID-19 related ARDS and is a potential biomarker for the disease severity [13, 14]. SARS-COV-2 may directly infect monocytes/ macrophages and induce tissue factor (TF) expression/release from these cells that may play a critical role in developing COVID-19 coagulopathy [15]. NETs used several pathways that support fibrin formation and enhance platelets activation and thrombosis. NETs can stimulate thrombosis in a platelet-dependent manner by adhesion and activation of platelets and binding the cells to von Willebrand factor (VWF) and fibrinogen or directly by coagulation cascade activation [16]. Neutrophils and platelets release microparticles that contain TF that NETs can trap. TF has been detected in NETs inside venous thrombi in vivo as a factor of induce thrombosis [17, 18]. NETs are involved in fibrinolysis inhibition by tissue plasminogen activator (tPA) inhibition [19]. Additionally NETs promote intrinsic coagulation cascade by activation of FXII by nucleic acids and phosphates and increase fibrin formation. NETs induce tissue factor pathway inhibitor (TFPI) degradation as the main source of extrinsic pathway inhibitor and increase the chance of blood coagulation [20].

### NETosis

NETosis/ETosis described as a form of necrosis that is associated with neutrophils (NETosis) and other granulocytes or macrophages (ETosis). In sepsis, after neutrophil stimulation with IL-8, lipopolysaccharide (LPS), TNF-α, and complement the decondensed chromatin networks or NETs are released by neutrophils [6–8]. Additionally, TLR2/4 and complement are responsible for initiating NETs formation and associated with dysregulated innate immune response and subsequent tissue injury and organ dysfunction [21].

**Fig. 1:**
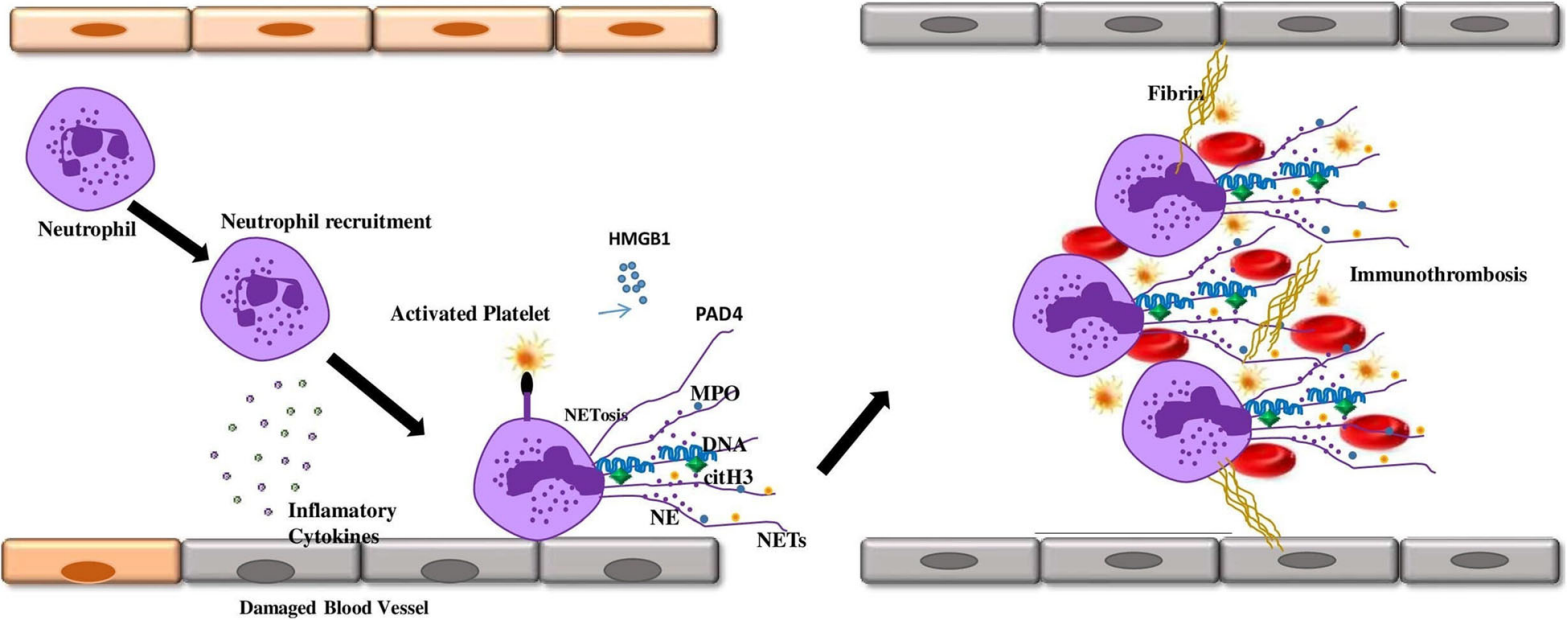
NETs formation can lead to vascular endothelial cells dysfunction. NETs contain decondensed chromatin, citrullinated-histones and proteolytic peptides such as myeloperoxidase (MPO), neutrophil elastase (NE) and high mobility group protein B1 (HMGB1), peptidyl arginine deaminase 4 (PAD4). NETs interaction to neutrophils and platelets lead to immunothrombosis formation

In the NETosis process, neutrophil granules contents translocate to the nucleus, decondense the chromatin, and induce a neutrophil extracellular trap (NETs). In NETosis, the internal, granular, and nuclear membranes are broken down and the cell content extruded to the extracellular space, but cytoplasmic membrane integrity is maintained [22]. NETs contain decondensed chromatin, histones, and proteolytic peptides such as myeloperoxidase (MPO), neutrophil elastase (NE), high mobility group protein B1 (HMGB1), matrix metalloproteinases (MMPs), proteinase 3 (PR3), and cathepsins that trap, immobilize, and kill microorganisms and activate other immune cells. Additionally, Peptidyl arginine deaminase 4 (PAD4) is transferred from the cytoplasm into the nucleus to catalyze citrullination of histones, leading to decondensation of chromatin. Viral infections stimulate NETs formation (Fig. 1). Virus-induced NETs can lead to hyperactivation of immune system response and produce cytokines, chemokines, immune complexes, and inflammation [22–25].

In vivo, plasma DNases degrade NETs and are subsequently cleared by macrophages [26, 27]. The NETs clearance importance is demonstrated in deficient DNase 1 and DNase 3 mice models. After a few days of inducing neutrophil activation, vessel occlusion occurred due to a large number of NETs, leading to death in the models.

### NETosis and intrinsic and extrinsic coagulation cascade

The coagulation cascade can be activated via extrinsic or intrinsic pathways. The extrinsic pathway is started by tissue factor (TF), which is normally expressed as a transmembrane protein in cells that are not directly in contact with the blood flow. When a blood vessel is damaged, TF is exposed to the blood and attaches to factor VII that is presented in blood and starts a common coagulation pathway. Activation of factor FXII initiates the intrinsic pathway upon binding to negatively charged surfaces, such as collagen and phospholipid. Subsequently, cleavage of FXI and FIX can use active FX and lead to fibrin formation [28].

Plasmin degraded fibrin is the important regulator preventing clot formation and thrombosis. Fibrin clots like NETs can capture bacteria and prevent their invasion. In contrast to fibrin clots, NETs contain antimicrobial proteins that enable them to prevent the pathogens from spreading and kill them [9, 29]. In an experiment in which whole blood was contained NETs, platelets recruited to NETs [10]. The platelets recruitment may be due to binding to C3b deposits on NETs because platelets express complement receptor 1(CR1) on their membrane and or mediated by histones [10, 30, 31]. NETs might lead to platelet aggregation as an essential step in clots formation. NETs contain histones, especially the type of H4 can activate platelets. Subsequently, the activated platelets releaseHMGB1that stimulate NETosis in a positive feedback loop. Additionally, histones stimulate platelets to secrete polyP from α-granules that activate FXII and blood coagulation's intrinsic pathway [32, 33]. Decorated NETs with platelets may be suitable scaffolds for thrombus formation. In this regard, experiments on NETs in mice treated with DNase showed the formation of smaller thrombi [34, 35]. The exact mechanism of coagulation activation by NETs remains unknown, and the separate components of NETs, including DNA and histones, have been demonstrated to induce thrombin formation [32]. Experiments demonstrated that neutrophils treated with cytokines can upregulate TF mRNA and release TF on their NETs, resulting in thrombin formation [36]. However, TF exposure on NETs apparently depends on the type of stimulation used to induce NETosis. Consequently, not all experiments have been shown NETs could produce TF and initiate coagulation [9]. NETs can directly activate intrinsic coagulation cascade because NETs have negative charges and could bind and activate FXII and induce thrombin generation [37]. In this regard, an in vitro study showed that inhibition of FXII or FXI in NETs reduced thrombin formation [38]. So that NETs can start the initiation of coagulation via either intrinsic or extrinsic pathways[9]. NE and, to a minor extent, cathepsin G as the NET-associated protein may contribute to fibrin formation on NETs because they could degrade TFPI. TFPI is the major extrinsic coagulation pathway inhibitor that recruited through nucleosome into the site of injury. [39] The fibrin clots mixed with NETs have more resistant to fibrinolysis by plasmin that might be a critical point in NETs related thrombosis. [40].

### NETosis and immune complexes

A major responsible factor to immunothrombosis development, a feature observed in COVID-19 infection that induces ARDS and disease severity, is NETs formation. A well-known mechanism for NETs formation is stimulation of the FcγRIIA and downstream SYK signaling pathway [41]. Immune complexes are the primary mediators of FcγRIIA signaling and NETosis. COVID-19 patients with robust antibody responses have been associated with poor clinical outcomes that raise the possibility of the contribution of immune complexes in NETs formation [42, 43]. Additionally, endogenous stimuli such as inflammatory cytokines, damage-associated molecular patterns (DAMPs), and pathogen-associated molecular patterns (PAMPs) could stimulate the release of NETs and the contribution of their role in COVID-19 infection remains to be clarified [44].

### NETosis and Factor Seven Activating Protease (FSAP) activation

Hyaluronan-binding protein 2 or factor VII activating protease is a serine protease produced by the liver, kidney, and pancreas. It is present in the circulation in the form of zymogen (pro-FSAP). It is known to activate coagulation factor-VII independent tissue factor and urokinase single-chain plasminogen activator[45, 46]. Several in vitro and patient-based studies have been shown a link between the levels of FSAP, inflammation, and disease. FSAP level is elevated in lung endothelial cells of acute lung injury induced by lipopolysaccharide and in the lungs of patients with acute respiratory distress syndrome [47–49]. In pathologic situations like acute coronary disease, ischemic stroke, and symptomatic carotid stenosis, FSAP levels increased. In addition, FSAP can activate inflammation pathways in non-immune cells like smooth muscle and endothelial cells and myeloid cells through NF-kB mediated proinflammatory cytokine production [50–52]. Elevated FSAP levels may indicate systemic inflammation that increases the risk of thrombosis in the COVID-19 patients.

### NETs and antiphospholipid antibodies

NETs and their associated components are known to stimulate thrombosis because intravascular NETosis can initiate thrombotic events in arteries, veins, and especially microvasculature and has a critical role in thrombosis formation in COVID-19 [53]. Antiphospholipid antibodies (aPLs) are known as one of the mechanisms of thrombosis through NETs formation. APLs promote NETs release in a manner dependent on Reactive oxygen species (ROS) and TLR4 [54]. NETs are essential in antiphospholipid syndrome (APS) because in APS, neutrophils are prone to spontaneous release of NETs. NETs are an essential activator of the coagulation cascade in APS [55]. NETs in APS are the main source of tissue factors (TFs), platelet activation, and aggregation and play a vital role in forming atherosclerosis and arterial thrombosis. In APS, neutrophils seem to have a greater adhesion potential, enhancing neutrophil-endothelium interaction and NETs diffusion [56]. Thus, neutrophils and NETosis are directly or indirectly involved in APS pathogenesis [57]. Many studies have reported low/moderate aPL titers in COVID-19 patients. These antibodies mainly target β2GP1 but indicate epitope characteristics different from APS antibodies [58, 59]. There is currently limited information on the role and importance of aPLs in COVID-19 pathogenesis, and evidence suggests that aPLs may have little clinical association with prolonged activated partial-thromboplastin time (aPTT) and thrombosis in COVID-19 patients [60, 61].

### NETosis inhibition

NETosis stimulates coagulation activation and fibrinolysis inhibition in various pathways. Targeting NETs formation might be a feasible and valuable therapeutic strategy to prevent thrombus formation and improve clinical outcomes of COVID-19 infection. Anti-cytokine therapy against IL-1β is widely used in various inflammatory and autoimmune diseases, preventing activation and accumulation of neutrophils and subsequently NETosis. The recombinant anakinra protein as IL-1β receptor antagonist may be a potential target to COVID-19 treatment and is currently undergoing clinical trials (https://clinicaltrials.gov: NCT04324021, NCT04330638, NCT02735707). Additionally, using inhibitors that target involved components of NETs formation process like NE, PAD4, and gasdermin D protein (GSDMD) during inflammation can be another approach. The NETosis prevention by nocodazole as a microtubule inhibitor is reported in in vitro experiments [62]. In this regard, clinical trials (https://clinicaltrials.gov: NCT04326790, NCT04328480, NCT04322565, NCT04322682) that test the efficacy of colchicine against COVID-19 are currently underway [63]. Drugs inhibitors of the NETosis axis such as glucocorticoids can block neutrophils' function and prevent NETs formation. Additionally, using exogenous DNase treatment improves NETs clearance, and recombinant human DNase I is currently under investigation for safety and efficacy in clinical trials in COVID-19.

## Conclusion

NETs formation in COVID-19 infection that is related to dysregulated immune response, releasing of inflammatory cytokines, and development of pathogenic microvascular thrombi. COVID-19 patients seem especially prone to excessive NETs formation and disease severity parallel increasing markers of NETosis [13, 64]. In this regard NETs, inhibitors may dampen the severity of SARS-COV-2 infection.

## Data Availability

Not applicable.

## Abbreviations

SARS-CoV-2: Severe acute respiratory syndrome coronavirus 2
COVID19: Coronavirus disease
TF: Tissue factor
NETs: Neutrophil extracellular traps
FSAP: Factor seven activating protease
TFPI: Tissue factor pathway inhibitor
ARDS: Acute respiratory distress syndrome
ACE2: Angiotensin converting enzyme

## References

[1] Leisman DE, Deutschman CS, Legrand M (2020). Facing COVID-19 in the ICU: vascular dysfunction, thrombosis, and dysregulated inflammation. Intensive Care Medicine.

[2] Pons S, Fodil S, Azoulay E, Zafrani L (2020). The vascular endothelium: the cornerstone of organ dysfunction in severe SARS-CoV-2 infection. Critical care.

[3] Rovas A, Osiaevi I, Buscher K, Sackarnd J, Tepasse P-R, Fobker M (2021). Microvascular dysfunction in COVID-19: the MYSTIC study. Angiogenesis.

[4] Geisbert TW, Young HA, Jahrling PB, Davis KJ, Kagan E, Hensley LE (2003). Mechanisms underlying coagulation abnormalities in ebola hemorrhagic fever: overexpression of tissue factor in primate monocytes/macrophages is a key event. The Journal of infectious diseases.

[5] Chan LL, Nicholls JM, Peiris JM, Lau YL, Chan MC, Chan RW (2020). Host DNA released by NETosis in neutrophils exposed to seasonal H1N1 and highly pathogenic H5N1 influenza viruses. Respiratory research.

[6] Vorobjeva N, Pinegin B (2014). Neutrophil extracellular traps: mechanisms of formation and role in health and disease. Biochemistry (Moscow).

[7] Ravindran M, Khan MA, Palaniyar N (2019). Neutrophil extracellular trap formation: physiology, pathology, and pharmacology. Biomolecules.

[8] Yousefi S, Simon D, Stojkov D, Karsonova A, Karaulov A, Simon H-U (2020). In vivo evidence for extracellular DNA trap formation. Cell death & disease.

[9] de Bont CM, Boelens WC, Pruijn GJ (2019). NETosis, complement, and coagulation: a triangular relationship. Cellular & molecular immunology.

[10] Fuchs TA, Brill A, Duerschmied D, Schatzberg D, Monestier M, Myers DD (2010). Extracellular DNA traps promote thrombosis. Proceed Natl Acad Sci..

[11] Czaikoski PG, Mota JMSC, Nascimento DC, Sônego F, Castanheira FVeS, Melo PH (2016). Neutrophil extracellular traps induce organ damage during experimental and clinical sepsis. PloS one.

[12] Lefrançais E, Mallavia B, Zhuo H, Calfee CS, Looney MR. Maladaptive role of neutrophil extracellular traps in pathogen-induced lung injury. JCI insight. 2018;3(3). 10.1172/jci.insight.98178 PMC5821185 29415887

[13] Zuo Y, Yalavarthi S, Shi H, Gockman K, Zuo M, Madison JA, et al. Neutrophil extracellular traps in COVID-19. JCI insight. 2020;5(11). 10.1172/jci.insight.138999 PMC7308057 32329756

[14] Barnes BJ, Adrover JM, Baxter-Stoltzfus A, Borczuk A, Cools-Lartigue J, Crawford JM, et al. Targeting potential drivers of COVID-19: Neutrophil extracellular traps. Journal of Experimental Medicine. 2020;217(6). 10.1084/jem.20200652 PMC7161085 32302401

[15] Eslamifar Z, Behzadifard M, Soleimani M, Behzadifard S (2020). Coagulation abnormalities in SARS-CoV-2 infection: overexpression tissue factor. Thrombosis journal.

[16] Martinod K, Wagner DD (2014). Thrombosis: tangled up in NETs. Blood, The Journal of the American Society of Hematology.

[17] Maroney SA, Mast AE (2008). Expression of tissue factor pathway inhibitor by endothelial cells and platelets. Transfusion and Apheresis Science.

[18] Novotny WF, Girard TJ, Miletich JP, Broze Jr GJ. Platelets secrete a coagulation inhibitor functionally and antigenically similar to the lipoprotein associated coagulation inhibitor. Blood. 1988;72(6):2020–5. 3143429

[19] Miesbach W (2020). Pathological role of angiotensin II in severe COVID-19. TH open.

[20] Broze GJ Jr, Miletich JP. Characterization of the inhibition of tissue factor in serum. Blood. 1987;69(1)150–5. 3024756

[21] Clark SR, Ma AC, Tavener SA, McDonald B, Goodarzi Z, Kelly MM (2007). Platelet TLR4 activates neutrophil extracellular traps to ensnare bacteria in septic blood. Nature medicine.

[22] Mozzini C, Girelli D (2020). The role of Neutrophil Extracellular Traps in Covid-19: Only an hypothesis or a potential new field of research?. Thrombosis research.

[23] Bonaventura A, Liberale L, Carbone F, Vecchié A, Diaz-Cañestro C, Camici GG (2018). The pathophysiological role of neutrophil extracellular traps in inflammatory diseases. Thrombosis and haemostasis.

[24] Bardoel BW, Kenny EF, Sollberger G, Zychlinsky A (2014). The balancing act of neutrophils. Cell host & microbe.

[25] Brinkmann V, Reichard U, Goosmann C, Fauler B, Uhlemann Y, Weiss DS (2004). Neutrophil extracellular traps kill bacteria. science.

[26] Jiménez-Alcázar M, Rangaswamy C, Panda R, Bitterling J, Simsek YJ, Long AT (2017). Host DNases prevent vascular occlusion by neutrophil extracellular traps. Science.

[27] Farrera C, Fadeel B (2013). Macrophage clearance of neutrophil extracellular traps is a silent process. The Journal of Immunology.

[28] Esmon CT (1987). The regulation of natural anticoagulant pathways. Science.

[29] Schulz C, Engelmann B, Massberg S (2013). Crossroads of coagulation and innate immunity: the case of deep vein thrombosis. Journal of thrombosis and haemostasis.

[30] Hamzeh-Cognasse H, Damien P, Chabert A, Pozzetto B, Cognasse F, Garraud O (2015). Platelets and infections–complex interactions with bacteria. Frontiers in immunology.

[31] van der Maten E, de Bont CM, de Groot R, de Jonge MI, Langereis JD, van der Flier M (2016). Alternative pathway regulation by factor H modulates Streptococcus pneumoniae induced proinflammatory cytokine responses by decreasing C5a receptor crosstalk. Cytokine.

[32] Noubouossie DF, Whelihan MF, Yu Y-B, Sparkenbaugh E, Pawlinski R, Monroe DM (2017). In vitro activation of coagulation by human neutrophil DNA and histone proteins but not neutrophil extracellular traps. Blood, The Journal of the American Society of Hematology.

[33] Semeraro F, Ammollo CT, Morrissey JH, Dale GL, Friese P, Esmon NL (2011). Extracellular histones promote thrombin generation through platelet-dependent mechanisms: involvement of platelet TLR2 and TLR4. Blood. The Journal of the American Society of Hematology.

[34] Brill A, Fuchs T, Savchenko A, Thomas G, Martinod K, De Meyer S (2012). Neutrophil extracellular traps promote deep vein thrombosis in mice. Journal of Thrombosis and Haemostasis.

[35] McDonald B, Davis RP, Kim S-J, Tse M, Esmon CT, Kolaczkowska E (2017). Platelets and neutrophil extracellular traps collaborate to promote intravascular coagulation during sepsis in mice. Blood, The Journal of the American Society of Hematology.

[36] Kambas K, Mitroulis I, Apostolidou E, Girod A, Chrysanthopoulou A, Pneumatikos I, et al. Autophagy mediates the delivery of thrombogenic tissue factor to neutrophil extracellular traps in human sepsis. 2012;e45427. 10.1371/journal.pone.0045427 PMC3446899 23029002

[37] von Brühl M-L, Stark K, Steinhart A, Chandraratne S, Konrad I, Lorenz M (2012). Monocytes, neutrophils, and platelets cooperate to initiate and propagate venous thrombosis in mice in vivo. Journal of Experimental Medicine.

[38] Gould TJ, Vu TT, Swystun LL, Dwivedi DJ, Mai SH, Weitz JI (2014). Neutrophil extracellular traps promote thrombin generation through platelet-dependent and platelet-independent mechanisms. Arteriosclerosis, thrombosis, and vascular biology.

[39] Massberg S, Grahl L, von Bruehl M-L, Manukyan D, Pfeiler S, Goosmann C (2010). Reciprocal coupling of coagulation and innate immunity via neutrophil serine proteases. Nature medicine.

[40] Thammavongsa V, Kim HK, Missiakas D, Schneewind O (2015). Staphylococcal manipulation of host immune responses. Nature Reviews Microbiology.

[41] Perdomo J, Leung HH, Ahmadi Z, Yan F, Chong JJ, Passam FH (2019). Neutrophil activation and NETosis are the major drivers of thrombosis in heparin-induced thrombocytopenia. Nature communications.

[42] Wang Y, Zhang L, Sang L, Ye F, Ruan S, Zhong B (2020). Kinetics of viral load and antibody response in relation to COVID-19 severity. The Journal of clinical investigation.

[43] Liu L, Wei Q, Lin Q, Fang J, Wang H, Kwok H, et al. Anti–spike IgG causes severe acute lung injury by skewing macrophage responses during acute SARS-CoV infection. JCI insight. 2019;4(4). 10.1172/jci.insight.123158 PMC6478436 30830861

[44] Papayannopoulos V (2018). Neutrophil extracellular traps in immunity and disease. Nature Reviews Immunology.

[45] Choi-Miura N-H, Tobe T, Sumiya J-i, Nakano Y, Sano Y, Mazda T (1996). Purification and characterization of a novel hyaluronan-binding protein (PHBP) from human plasma: it has three EGF, a kringle and a serine protease domain, similar to hepatocyte growth factor activator. The journal of biochemistry.

[46] Römisch J, Feussner A, Vermöhlen S, Stöhr H (1999). A protease isolated from human plasma activating factor VII independent of tissue factor. Blood coagulation & fibrinolysis: an international journal in haemostasis and thrombosis.

[47] Mambetsariev N, Mirzapoiazova T, Mambetsariev B, Sammani S, Lennon FE, Garcia JG (2010). Hyaluronic acid binding protein 2 is a novel regulator of vascular integrity. Arteriosclerosis Thrombosis Vasc Biol..

[48] Ware LB, Matthay MA (2000). The acute respiratory distress syndrome. New England Journal of Medicine.

[49] Wygrecka M, Markart P, Fink L, Guenther A, Preissner KT (2007). Raised protein levels and altered cellular expression of factor VII activating protease (FSAP) in the lungs of patients with acute respiratory distress syndrome (ARDS). Thorax.

[50] Parahuleva MS, Hölschermann H, Zandt D, Pons-Kühnemann J, Parviz B, Weiskirchen R, et al. Circulating factor VII activating protease (FSAP) is associated with clinical outcome in acute coronary syndrome. Circulation Journal. 2012:CJ-11-1502. 10.1253/circj.cj-11-1502 22850287

[51] Hanson E, Kanse S, Joshi A, Jood K, Nilsson S, Blomstrand C (2012). Plasma factor VII-activating protease antigen levels and activity are increased in ischemic stroke. Journal of thrombosis and haemostasis.

[52] Byskov K, Boettger T, Ruehle PF, Nielsen NV, Etscheid M, Kanse SM (2017). Factor VII activating protease (FSAP) regulates the expression of inflammatory genes in vascular smooth muscle and endothelial cells. Atherosclerosis.

[53] Wang Y, Luo L, Braun OÖ, Westman J, Madhi R, Herwald H (2018). Neutrophil extracellular trap-microparticle complexes enhance thrombin generation via the intrinsic pathway of coagulation in mice. Scientific reports.

[54] Jariwala MP, Laxer RM (2021). NETosis in rheumatic diseases. Current rheumatology reports.

[55] Tambralli A, Gockman K, Knight JS (2020). NETs in APS: current knowledge and future perspectives. Current Rheumatology Reports.

[56] Zuo Y, Shi H, Li C, Knight JS (2020). Antiphospholipid syndrome: a clinical perspective. Chinese Medical Journal.

[57] Bruschi M, Petretto A, Bertelli R, Galetti M, Bonanni A, Pratesi F (2017). Post-translational modified proteins are biomarkers of autoimmune-processes: NETosis and the inflammatory–autoimmunity connection. Clinica chimica acta.

[58] Borghi MO, Beltagy A, Garrafa E, Curreli D, Cecchini G, Bodio C, et al. Anti-phospholipid antibodies in COVID-19 are different from those detectable in the anti-phospholipid syndrome. Frontiers in immunology. 2020:2692. 10.3389/fimmu.2020.584241 PMC7593765 33178218

[59] Xiao M, Zhang Y, Zhang S, Qin X, Xia P, Cao W (2020). Antiphospholipid antibodies in critically Ill patients with COVID-19. Arthritis & Rheumatology.

[60] Mehta S, Bhandari S, Mehta S (2020). Cautious interpretation of antiphospholipid antibodies in COVID-19. Clinica Chimica Acta. International Journal of Clinical Chemistry.

[61] Galeano-Valle F, Oblitas C, Ferreiro-Mazón M, Alonso-Muñoz J, Del Toro-Cervera J, Di Natale M (2020). Antiphospholipid antibodies are not elevated in patients with severe COVID-19 pneumonia and venous thromboembolism. Thrombosis research.

[62] Neeli I, Dwivedi N, Khan S, Radic M (2009). Regulation of extracellular chromatin release from neutrophils. Journal of innate immunity.

[63] Vorobjeva N, Chernyak B (2020). NETosis: molecular mechanisms, role in physiology and pathology. Biochemistry (Moscow).

[64] Middleton EA, He X-Y, Denorme F, Campbell RA, Ng D, Salvatore SP (2020). Neutrophil extracellular traps contribute to immunothrombosis in COVID-19 acute respiratory distress syndrome. Blood.

